# Intra-session absolute and relative reliability of pressure pain thresholds in the low back region of vine-workers: effect of the number of trials

**DOI:** 10.1186/s12891-016-1212-7

**Published:** 2016-08-18

**Authors:** Romain Balaguier, Pascal Madeleine, Nicolas Vuillerme

**Affiliations:** 1 Université Grenoble-Alpes, EA AGEIS, La Tronche, France; 2 Physical Activity and Human Performance group - SMI, Department of Health Science and Technology, Aalborg University, Aalborg, Denmark; 3 Institut Universitaire de France, Paris, France

**Keywords:** Agriculture, Low back pain, Vineyard workers, Pressure pain topography, Reliability

## Abstract

**Background:**

Pressure pain thresholds (PPT) are commonly used to quantify mechanical pain sensitivity of deep structures. Excellent PPT reliability has been previously reported among the low back of healthy subjects. However, there is a lack of studies assessing PPT over the low back of workers exposed to biomechanical risk factors of low back pain. Thus, the purpose of this study was threefold: (1) to evaluate the intra-session absolute and relative reliability as well as minimal detectable change (MDC) values of PPT within 14 locations covering the low back region of vine-workers and (2) to determine the number of trial required to ensure reliable PPT assessments and (3) to assess the effect of modifier factors such as gender, age, body mass index (BMI) and pain intensity on PPT reliability.

**Methods:**

Twenty-nine vine-workers voluntarily participated in this study. Twenty-two reported low intensity of low-back pain while seven were pain-free. PPTs were assessed among 14 anatomical locations in the lower back region. Three trials were performed on each location with an interval time of at least one minute. Reliability was assessed computing intraclass correlation coefficients (ICC), standard error of measurement (SEM) for all possible combinations between trials. Bland-Altman plots were also generated to assess potential bias in the dataset. Finally, a repeated measure analysis of variance (RM-ANOVA) with the number of trials used as within subject factor was performed on (1) PPT, (2) ICC and (3) SEM values.

**Results:**

ICC ranged from 0.86 to 0.99 for all anatomical locations and for all possible combinations between trials. SEM for comparison between trial 1–2, 2–3, 1–3 and, 1-2-3 ranged from respectively, 36.7–77.5, 27.8–77.7, 50–95.2 and, 39.3–80.8 kPa. ICC and SEM remained similar to the ones obtained for the entire population when taking modifier factors in consideration. The visual analysis of Bland-Altman plots suggested small measurement errors for all anatomical locations and for all possible combinations between trials.

**Conclusions:**

The assessment of PPTs of the lower back among vine-workers was found to have excellent relative and absolute reliability. Moreover, reliable measurements can be equally achieved when using the mean of three PPT measurement or with the first one.

## Background

Work-related Musculoskeletal Disorders (WMSDs) are considered in numerous countries as a public health problem [1, 2]. WMSDs are often accompanied by pain located in the low back region [3, 4] and associated with muscle hyperalgesia [5]. Seventy percent of the population will experience low back pain at least once in its lifetime [6–8]. In France, the prevalence of low back pain is particularly high especially in viticulture partly explained by the relatively high exposure to biomechanical risk factors, i.e. awkward postures, repetitiveness [9, 10].

In many studies dealing with WMSDs, visual analogue scale (VAS) and numeric rating scale (NRS) are commonly used to measure pain intensity in the low back region [11, 12]. Even if these self-reported methods of pain intensity are considered valid, reliable and responsive to change in the intensity of pain [13], they are also largely influenced by psychosocial aspects related to the environment and the beliefs concerning the expected duration of pain [14]. However, pain sensitivity is not uniformly distributed in a body region or along a muscle [15–18]. Using pain diagram to depict painful areas does not in general offer the possibility to visualize the spatial distribution of pain sensitivity [19].

Assessing pressure pain threshold (PPT) is a way of quantifying sensitivity of deep structures to mechanical pain [20, 21]. PPT has a good to excellent relative reliability in many anatomical locations such as neck [22–24], knee [25], temporalis and masseter muscles [26, 27] and the low back region [28–31]. However, for this latter anatomical location, only few studies assessed PPT’s absolute reliability [31–35]. While topographical pain sensitivity mapping technique has been developed in the low back [36–38], the number of assessed points in studies dealing with PPT’s reliability in the low back is limited to two locations (2 cm laterally from L3 or L4 spinal processes). Moreover, most of these studies assessed PPT on young healthy subjects [31] and little is known about PPT’s reliability among workers exposed to biomechanical risk factors of WMSDs in the low back region. Consequently, it is essential to have reliable tools assessing mechanical sensitivity to pain in order to e.g. monitor the effectiveness of an intervention among workers with occupations potentially leading to WMSDs like vine-workers. This need is further substantiated by the difference found in PPT when comparing workers to young asymptomatic individuals underlining mechanical hyperalgesia [5, 39, 40].

The purpose of this study was threefold: (1) to evaluate the intra-session absolute and relative reliability as well as minimal detectable change (MDC) values of PPT within 14 locations covering the low back region of vine-workers and (2) to determine the number of trial required to ensure reliable PPT assessments and (3) to assess the effect of modifier factors such as gender, age, body mass index (BMI) and pain intensity on PPT reliability.

## Methods

### Participants

Twenty-nine adult vine-workers (16 men and 13 women) volunteered to participate in this study. Nineteen vine-workers out of 29 reported low-back pain at least 3 consecutive days in the last 12 months and seven were pain free at the time of measurements. The workers were recruited from two vineyards in the Bordeaux (France) wine district. The vine-workers’ characteristics (anthropometrics, low-back pain duration and intensity) are presented in Table 1. The inclusion criteria were: age between 25 and 60 years, full-time employed as vine-worker, no history of spine or pelvis fracture, no tumor or spinal surgery and no pregnancy.

**Table 1:** Characteristics of the vine-workers

	Mean (standard deviation)
Age (years)	39.9 (9.9)
Height (cm)	168.7 (8.6)
Body mass (kg)	77.3 (17.5)
Body mass index (kg/m^2^)	27.2 (0.24)
Job seniority (years)	17 (9)
Back Pain > 3 days in the last 12 months	19 out of 26
Average LBP (NRS 0–10) in the last 7 days	2.7 (2.4)

### Experimental protocol

The PPT measurements were performed during working hours in one session lasting approx. 30 min using a hand-held electronic algometer (Somedic Algometer type 2, Sollentuna, Sweden) with a 1 cm^2^ wide rubber tip. PPTs were assessed over 14 anatomical locations in the low back region with 7 locations on each side of the lumbar spinal processes L1-L5. Each location was measured trice (Trial 1, Trial 2 and Trial 3) by a single rater with at least 1 min between two consecutive trials on the same location to avoid temporal sensitization [41]. The algometer was calibrated prior to data collection. Pressure was applied at a constant slope of 30 kPa/s with the tip of the algometer perpendicular to the skin. The worker was lying comfortably in a prone position on a table and was asked to press a button that locks the algometer display when the feeling of pressure changed to pain. Then, the examiner noted the pressure indicated on the algometer display corresponding to the PPT. Prior to recordings, the worker was familiarized with PPT assessment by measuring PPT on the tibialis anterior muscle considered as a reference point [42, 43].

### Procedure to mark the 14 anatomical locations

**Fig. 1:**
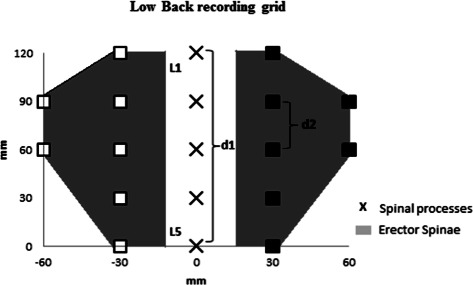
Schematic representation of the low back pressure pain threshold recording grid of the left (blank square) and right (black squares) erector spinae muscles. d1 represents the distance between the first (L1) and the fifth (L5) lumbar vertebrae. d2 equals one fourth of d1

Eight paper grids were designed for PPT measurements in the low back region. The design was based on the studies by Binderup and colleagues [36, 37] where the distance between two adjacent locations is calculated from the distance (d1) between L1 and L5. Then, we calculated the quarter of this distance (d2). A first column of 5 points was placed bilaterally at the distance (d2) from a fictive line joining L1 to L5. Then, a second column of 2 points was set bilaterally at 2 times the distance (d2) of L2 and L3 (Fig. 1). Binderup and colleagues [36] have shown that the distance L1-L5 is on average 14.3 ± 2.8 cm for adult men and 12.5 ± 0.9 cm for adult women. Based on these distances, we have developed eight PPT grids with a L1-L5 distance ranging from 11 to 14.5 cm (using step of 0.5 cm between two consecutive grids) [31]. The rater palpated the lumber spinal process L1 and L5 and placed a mark on the skin with a pencil on those two locations. Then, the distance L1-L5 was measured to select the corresponding PPT grid. Once selected, the rater aligned the grid with the L1 and L5 marks on the skin and started the assessments. The use of the designed grid results in a gain of time of approx. 20 min without alterations of the gain in spatial information.

### Data analysis

PPT measurements, intraclass correlation coefficients (ICC) and standard error of measurement (SEM) values were normally distributed (Shapiro-Wilk normality test). The magnitude of the systematic difference in PPT between trials was estimated using 95 % confidence interval (CI) of the mean difference (Mean_Diff_) between trials 1–2, trials 1–3, trials 2–3 and calculated with the formula:

$$ \mathrm{C}\mathrm{I}=\mathrm{MeanDiff}\pm {\mathrm{t}}_{\mathrm{n}}{{\textstyle \hbox{-}}}_1\times \sqrt{\left(\frac{\mathrm{SDDiff}}{\mathrm{n}}\right)} $$

where, t_n-1_ corresponds to the value of t distribution with n-1° of freedom where n corresponds to the number of participants. A repeated measure analysis of variance (RM-ANOVA) with the number of trials used as within subject factor was performed on (1) PPT, (2) ICC and (3) SEM values. In case of significant effect of the number of trials, a Tukey post-hoc for pair-wise comparisons test was used to compare differences between trials. The relative and absolute reliability across the trials 1-2-3 were computed using ICC, SEM and minimal detectable change (MDC). The relative reliability was evaluated by calculating a 2-way fixed ICC_2,1_ (for absolute agreement). ICC values were interpreted using the categories proposed previously in which an ICC between 0.00 and 0.20 is considered poor, 0.21–0.40 is fair, 0.41–0.60 is moderate, 0.61–0.80 is substantial, and 0.81–1.00 is almost perfect [44]. SEM is an absolute measure of the variability of the errors of measurement and allows making statement about the precision of test scores of individual examinees [45]. SEM has the same unit of measurement (kPa). According to Harvill [35], SEM was generated with the following formula:

$$ \mathrm{S}\mathrm{E}\mathrm{M}=\mathrm{S}\mathrm{D}\sqrt{1-ICC} $$

where SD is the standard deviation of the scores from all workers and ICC the relative reliability. MDC gives the minimum value for which a difference can be considered as “real”. MDC was calculated with the formula:

$$ \mathrm{M}\mathrm{D}\mathrm{C}=\mathrm{S}\mathrm{E}\mathrm{M}\times 1.96\times \sqrt{2} $$

Furthermore, Bland and Altman plots of the differences between trials against their mean and limits of agreements (LOA) were used to assess the magnitude of disagreement between trials. A difference between trials outside the LOA can be considered as a real change [46]. Additional analyses using gender and a median split for age, BMI and pain intensity were conducted for the PPTs from the overall low-back (mean PPTs of the 14 anatomical locations) to address the effects of modifier factors. A student *t*-test was then used to compare groups. All data analyses were performed with R 3.0.1 software. Results are presented as mean (SD) or (95 % confidence interval), unless otherwise indicated. *p* < 0.05 was considered significant.

## Results

### Intra-session relative and absolute reliability of PPT in the low back

The ICCs of the 14 anatomical locations and of the left, right and overall low-back (P_left_, P_right_, P_all_) were almost perfect regardless of the conducted comparison (trials 1-2-3). Likewise the absolute reliability,.i.e. SEM did not change significantly (Table 2). The ICC, SEM and MDC following a median split were similar to the ones obtained for the entire population (Table 3).

**Table 2:** Intraclass correlation coefficients (ICC), standard error of measurement (SEM) and minimum detectable change (MDC) for pressure pain thresholds assessed over 14 locations (P1 to P14) over the low back region, for left and right side (P_left_ and P_right_) as well as overall low back (P_all_) between the mean of the first and second trials (T1-T2), the first and the third trials (T1-T3), the second and the third trials (T2-T3) and the means of the three trials (T1-T2-T3)

Trials	T1-T2				T1-T3				T2-T3				T1-T2-T3			
Points	ICC (95 % CI)	SEM (kPa)	MDC (kPa)	MDC (%)	ICC (95 % CI)	SEM (kPa)	MDC (kPa)	MDC (%)	ICC (95 % CI)	SEM (kPa)	MDC (kPa)	MDC (%)	ICC (95 % CI)	SEM (kPa)	MDC (kPa)	MDC (%)
P1	0.92 (0.84–0.96)	65.9	182.7	38.1	0.88 (0.77–0.94)	82.9	229.7	48.2	0.92 (0.84–0.96)	66.7	185.0	39.0	0.91 (0.84–0.95)	72.0	199.7	41.9
P2	0.95 (0.90–0.98)	53.5	148.4	31.2	0.91 (0.83–0.96)	72.1	199.8	41.1	0.94 (0.88–0.97)	60.1	166.5	34.2	0.94 (0.89–0.97)	62.2	172.4	35.7
P3	0.92 (0.84–0.96)	72.8	201.9	42.2	0.89 (0.78–0.95)	86.6	240.1	48.9	0.96 (0.91–0.98)	54.0	149.8	31.1	0.92 (0.86–0.96)	72.2	200.1	41.4
P4	0.92 (0.83–0.96)	71.9	199.4	38.8	0.88 (0.77–0.94)	86.7	240.4	45.7	0.95 (0.89–0.97)	61.9	171.6	32.5	0.92 (0.85–0.96)	74.0	205.1	39.2
P5	0.91 (0.83–0.96)	75.6	209.4	41.5	0.86 (0.71–0.93)	95.2	263.8	52.3	0.93 (0.86–0.97)	70.4	195.1	38.4	0.90 (0.83–0.95)	80.8	224.0	44.3
P6	0.92 (0.84–0.96)	67.3	186.5	35.2	0.88 (0.76–0.94)	81.0	224.6	41.8	0.95 (0.90–0.98)	53.0	147.0	26.9	0.92 (0.85–0.96)	67.9	188.2	35.0
P7	0.93 (0.85–0.97)	65.8	182.3	37.3	0.89 (0.78–0.95)	77.2	213.9	43.9	0.92 (0.83–0.96)	71.0	196.8	39.1	0.91 (0.85–0.96)	71.3	197.6	40.1
P8	0.90 (0.80–0.95)	77.5	214.7	45.1	0.89 (0.77–0.95)	82.6	228.9	47.7	0.95 (0.89–0.97)	59.4	164.7	33.2	0.91 (0.85–0.96)	73.7	204.2	42.2
P9	0.94 (0.87–0.97)	65.6	181.8	35.7	0.91 (0.81–0.96)	74.7	207.2	39.9	0.95 (0.90–0.98)	57.4	159.1	30.1	0.93 (0.88–0.97)	66.1	183.3	35.3
P10	0.94 (0.87–0.97)	60.7	168.3	33.3	0.93 (0.86–0.97)	64.9	180.0	34.4	0.94 (0.87–0.97)	63.4	175.8	33.6	0.94 (0.89–0.97)	62.9	174.4	33.7
P11	0.94 (0.86–0.97)	61.8	171.2	32.5	0.94 (0.85–0.97)	62.2	172.3	32.7	0.92 (0.85–0.96)	67.8	188.0	34.4	0.93 (0.88–0.97)	63.8	176.9	33.2
P12	0.93 (0.86–0.97)	62.5	173.2	33.0	0.91 (0.82–0.96)	75.1	208.0	40.1	0.90 (0.80–0.95)	77.7	215.5	40.3	0.92 (0.85–0.96)	71.9	199.2	37.9
P13	0.95 (0.89–0.98)	55.6	154.1	29.4	0.94 (0.87–0.97)	58.6	162.4	30.4	0.93 (0.85–0.97)	66.0	183.1	33.8	0.94 (0.89–0.97)	60.1	166.5	31.2
P14	0.94 (0.88–0.97)	59.2	164.0	32.7	0.93 (0.80–0.97)	65.0	180.2	35.2	0.95 (0.90–0.98)	51.1	141.7	27.0	0.94 (0.89–0.97)	58.6	162.4	31.7
P_left_	0.97 (0.94–0.99)	38.3	106.3	21.3	0.95 (0.90–0.98)	50.6	140.3	27.8	0.99 (0.97–0.99)	27.8	77.0	15.0	0.97 (0.95–0.99)	39.9	110.6	21.9
P_right_	0.97 (0.94–0.99)	42.2	117.0	23.1	0.95 (0.89–0.98)	51.8	143.7	28.1	0.98 (0.95–0.95)	37.5	103.8	19.9	0.97 (0.94–0.98)	44.1	122.3	23.8
P_all_	0.98 (0.95–0.99)	36.7	101.8	20.2	0.95 (0.90–0.98)	50.0	138.7	27.3	0.99 (0.97–0.99)	28.3	78.5	15.2	0.97 (0.95–0.99)	39.3	108.9	21.4

**Table 3:** Intraclass correlation coefficients (ICC), standard error of measurement (SEM) and minimum detectable change (MDC) for pressure pain thresholds assessed over the overall low back P_all_ (representing the average of the 14 PPT assessments) between the mean of the first and second trials (T1-T2), the first and the third trials (T1-T3), the second and the third trials (T2-T3) and the means of the three trials (T1-T2-T3) using gender and a median split for age, BMI and, pain intensity

	T1-T2				T1-T3				T2-T3				T1-T2-T3			
	ICC (95 % CI)	SEM (kPa)	MDC (kPa)	MDC (%)	ICC (95 % CI)	SEM (kPa)	MDC (kPa)	MDC (%)	ICC (95 % CI)	SEM (kPa)	MDC (kPa)	MDC (%)	ICC (95 % CI)	SEM (kPa)	MDC (kPa)	MDC (%)
Men	0.98 (0.93–0.99)	35.8	99.2	17.3	0.95 (0.87–0.98)	50.1	138.9	24.1	0.98 (0.95–0.99)	30.6	84.9	14.5	0.97 (0.93–0.99)	39.5	109.5	18.9
Women	0.97 (0.91–0.99)	36.8	102.1	25.0	0.95 (0.86–0.98)	49.1	136.1	32.7	0.99 (0.97–1.00)	24.0	66.5	15.8	0.97 (0.93–0.99)	37.8	104.8	25.3
Age ≤ 41	0.97 (0.92–0.99)	36.4	101.0	20.3	0.96 (0.87–0.98)	47.7	132.3	26.3	0.99 (0.96–1.00)	23.4	65.0	13.2	0.97 (0.94–0.99)	37.0	102.5	20.5
Age > 41	0.98 (0.91–0.99)	35.9	99.5	19.8	0.96 (0.94–0.99)	51.8	143.6	28.3	0.98 (0.95–0.99)	32.0	88.7	17.0	0.97 (0.93–0.99)	40.6	112.5	22.1
BMI ≤ 25	0.95 (0.86–0.98)	42.7	118.3	25.0	0.91 (0.75–0.97)	56.2	155.7	32.6	0.97 (0.91–0.99)	34.5	95.6	19.9	0.95 (0.87–0.98)	45.1	124.9	26.2
BMI > 25	0.98 (0.96–1.00)	28.9	80.0	15.3	0.97 (0.91–0.99)	43.0	119.2	22.5	0.99 (0.98–0.99)	19.9	55.2	10.2	0.99 (0.96–1.00)	31.9	88.3	16.6
Pain ≤ 2.5	0.97 (0.87–0.99)	37.9	105.0	17.8	0.93 (0.79–0.97)	57.9	160.4	26.9	0.98 (0.88–0.99)	35.2	97.6	16.0	0.96 (0.90–0.99)	44.6	123.6	20.7
Pain > 2.5	0.97 (0.92–0.99)	34.5	95.6	23.0	0.96 (0.89–0.99)	40.5	112.4	26.6	0.99 (0.97–1.00)	18.7	51.7	12.4	0.97 (0.94–0.99)	32.4	89.7	21.4

**Fig. 2:**
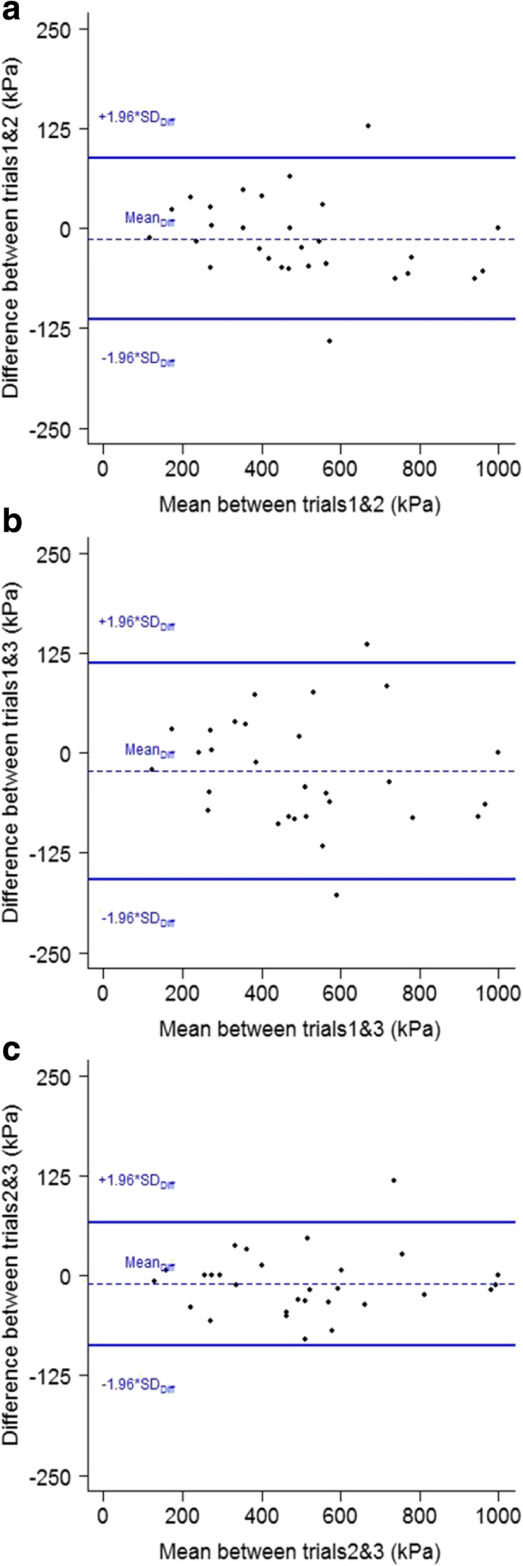
Bland and Altman analyses plotted for worker’s pressure pain threshold of the overall low back when the mean of the first and second trials **a**, the first and third trials **b** and the second and third trials **c** are considered

The visual analysis of the Bland-Altman plots (Fig. 2) suggested small measurement error whatever the comparison considered between trials. These plots also showed that zero was included in the 95 % confidence interval and that no apparent systematic bias was present in the data.

### Number of trials to ensure reliable measurements

The mean PPT values at each PPT location were not significantly different between trials regardless of the three conducted comparisons (trial 1 vs. trial 2, trial 1 vs. trial 3, trial 2 vs. trial 3), the p-values were ranged from 0.7457 to 1.000 (Tables 4, 5 and 6). Concerning the left, right and overall low-back (P_left_, P_right_, P_all_), p-values ranged from 0.8884 to 0.9994 (Tables 4 and 5). Lower PPT values were found for women compared with men for trial 1 and workers reporting pain intensity above 2.5 compared with workers reporting pain intensity below 2.5 for trial 2, trial 3 and the mean of the three trials (Table 5).

**Table 4:** Mean (standard deviation: SD) pressure pain thresholds (kPa) assessed over 14 locations (P1 to P14) covering the low back region, for left and right side (P_left_ and P_right_) as well as overall low back (P_all_) and level of significance (*p*-values) among trial. See “*Procedure to mark the 14 anatomical locations”* for explanation concerning the locations of PPT assessments

	Trial 1		Trial 2		Trial 3		T1-T2-T3		T1−T2	T1−T3	T2−T3
	Mean	(SD)	Mean	(SD)	Mean	(SD)	Mean	(SD)	*p*-value	*p*-value	*p*-value
P1	480.9	(242.6)	478.3	(237.4)	471.7	(245.0)	477.0	(238.8)	0.9991	0.9888	0.9942
P2	475.1	(241.4)	476.8	(246.4)	497.8	(255.9)	483.2	(245.2)	0.9997	0.9377	0.9462
P3	487.8	(263.4)	468.7	(259.5)	495.0	(264.7)	483.8	(259.6)	0.9598	0.9942	0.9253
P4	511.6	(236.4)	515.4	(269.6)	541.3	(273.1)	522.8	(257.4)	0.9984	0.9046	0.9265
P5	501.0	(241.9)	509.2	(276.3)	507.4	(262.6)	505.9	(257.5)	0.9923	0.9953	0.9996
P6	521.5	(228.1)	537.5	(247.5)	554.5	(240.2)	537.8	(236.2)	0.9661	0.8635	0.9616
P7	473.6	(231.4)	504.8	(264.0)	501.3	(238.7)	493.2	(242.5)	0.8828	0.9064	0.9984
P8	459.5	(236.5)	492.5	(261.2)	499.6	(259.5)	483.9	(250.2)	0.8766	0.8240	0.9940
P9	499.4	(250.0)	520.3	(273.7)	538.4	(250.6)	519.3	(255.7)	0.9505	0.8391	0.9631
P10	506.1	(244.0)	505.9	(252.1)	539.2	(260.4)	517.1	(249.7)	1.0000	0.8758	0.8745
P11	507.4	(255.2)	547.5	(253.3)	545.7	(244.2)	533.5	(248.6)	0.8213	0.8357	0.9996
P12	508.0	(246.2)	540.8	(242.6)	528.8	(258.8)	525.9	(246.6)	0.8750	0.9476	0.9823
P13	516.0	(245.4)	533.2	(246.6)	551.2	(249.0)	533.5	(244.5)	0.9632	0.8554	0.9600
P14	488.2	(244.8)	513.7	(249.4)	535.6	(233.5)	512.5	(240.5)	0.9184	0.7457	0.9390
P_left_	493.1	(232.2)	505.3	(241.0)	517.8	(242.7)	505.4	(236.0)	0.9800	0.9212	0.9793
P_right_	497.7	(235.2)	515.3	(254.5)	526.1	(244.2)	513.1	(242.1)	0.9609	0.9013	0.9850
P_all_	495.4	(232.7)	510.3	(246.2)	522.0	(242.2)	509.2	(237.8)	0.9708	0.9105	0.9821

**Table 5:** Mean (standard deviation: SD) pressure pain thresholds (kPa) assessed over the overall low back Pall (representing the average of the 14 PPT assessments) and level of significance (*p*-values) among trials using gender and a median split for age, body mass index (BMI) and, pain intensity. See “*Procedure to mark the 14 anatomical locations”* for explanation concerning the locations of PPT assessments

	Trial 1		Trial 2		Trial 3		T1-T2-T3		T1−T2	T1−T3	T2−T3
	Mean	(SD)	Mean	(SD)	Mean	(SD)	Mean	(SD)	*p*-value	*p*-value	*p*-value
Males	565.6*	230.0	580.7	238.2	587.3	231.9	587.3	231.9	0.9817	0.9627	0.9965
Females	403.3	201.1	413.7	223.8	428.1	228.3	415.0	212.5	0.9919	0.9547	0.9844
Age ≤ 41	498.6	227.7	495.8	231.5	506.0	230.7	500.1	224.7	0.9994	0.9957	0.9919
Age > 41	486.7	238.7	516.6	262.9	526.6	258.1	510.0	247.8	0.9479	0.9092	0.9940
BMI ≤ 25	470.6	197.9	474.2	204.9	484.0	187.9	476.3	192.3	0.9987	0.9823	0.9906
BMI > 25	513.7	259.7	535.3	277.7	545.8	283.7	531.6	267.9	0.9744	0.9449	0.9940
Pain ≤ 2.5	572.7	230.5	606.0*	237.0	613.4*	227.3	597.4*	226.6	0.9236	0.8884	0.9961
Pain > 2.5	418.3	207.7	412.4	215.6	425.0	221.2	418.6	210.0	0.9968	0.9960	0.9858

* significant difference between groups (*p* < 0.05)

**Table 6:** Mean difference between trials (95 % confidence interval (CI)) in pressure pain threshold (PPT, kPa) for the 14 locations (P1 to P14) covering the low back region, for left and right side (P_left_ and P_right_) as well as overall low back (P_all_). See “*Procedure to mark the 14 anatomical locations”* for explanation concerning the locations of PPT assessments

	Trial 1−Trial 2	Trial 1−Trial 3	Trial 2−Trial 3
	Mean PPT difference (95 % CI)	Mean PPT difference (95 % CI)	Mean PPT difference (95 % CI)
P1	2.6 (0.6 to 4.6)	9.2 (8.3 to 10.1)	6.6 (3.7 to 9.5)
P2	−1.6 (−3.6 to 0.3)	−22.6 (−28.2 to −17.1)	−21.0 (−24.6 to −17.4)
P3	19.1 (17.7 to 20.6)	−7.2 (−7.7 to −6.7)	−26.4 (−28.3 to −24.4)
P4	−3.8 (−16.4 to 8.8)	−29.7 (−43.7 to −15.7)	−25.9 (−27.3 to −24.5)
P5	−8.3 (−21.4 to 4.9)	−6.4 (−14.3 to 1.4)	1.8 (−3.4 to 7.1)
P6	−16.0 (−23.4 to −8.6)	−33.0 (−37.6 to −28.3)	−17.0 (−19.8 to −14.2)
P7	−31.2 (−43.6 to −18.8)	−27.7 (−30.5 to −24.9)	3.5 (−6.1 to 13.1)
P8	−33.0 (−42.5 to −23.6)	−40.1 (−48.8 to - 31.3)	−7.0 (−7.7 to −6.4)
P9	−21.0 (−30.0 to −11.9)	−39.0 (−39.2 to −38.8)	−18.0 (−26.9 to −9.2)
P10	0.2 (−2.9 to 3.3)	−33.1 (−39.4 to −26.9)	−33.3 (−36.5 to −30.1)
P11	−40.1 (−40.9 to −39.4)	−38.3 (−42.5 to −34.1)	1.8 (−1.6 to 5.3)
P12	−32.8 (−34.2 to −31.5)	−20.8 (−25.6 to −16.0)	12.0 (5.8 to 18.2)
P13	−17.2 (−17.7 to 16.8)	−35.2 (−36.5 to −33.8)	−18.0 (−18.9 to −17.0)
P14	−25.5 (−27.3 to −23.7)	−47.4 (−36.5 to −33.8)	−21.9 (−28.0 to −15.9)
P_left_	−12.2 (−15.5 to −8.9)	−24.6 (−28.6 to −20.6)	−12.4 (−13.1 to −11.8)
P_right_	−17.6 (−24.9 to −10.3)	−28.4 (−31.9 to −25.0)	−10.8 (−14.7 to −6.9)
P_all_	−14.9 (−20.0 to −9.8)	−26.5 (−30.2 to −22.9)	−11.6 (−13.1 to −10.1)

The comparison of means of ICC regardless of the conducted comparisons between trials showed statistical differences for Trials 1,3 vs. Trials 1,2 (*p* = 0.0171) and for Trials 2,3 vs. Trials 1,3 (*p* = 0.0009). The same analysis for SEM values further showed a statistical difference for the comparison Trials 2,3 vs. Trials 1,3 (*p* = 0.0122).

## Discussion

The purposes of this study were (1) to evaluate the intra-session absolute and relative reliability as well as MDC values of PPT assessments in the low back region (2) to determine the number of trial recordings required to ensure reliable PPT measurements among vine-workers and (3) to assess the effect of modifier factors such as gender, age, BMI and pain intensity on PPT reliability. This study particularly targeted a population at high risk of developing WMSDs i.e., vine-workers and used pressure pain sensitivity maps of the lumbar region.

Approximately 66 % of the workers reported episode of back pain lasting for more than 3 days within the last year. The low back pain intensity within the last 7 days was low. These self-reported values confirmed that low back pain is an issue among vine-workers. Compared to other workers, the PPT values of vine workers were close to those observed among cleaners and elderly administrative or nursing workers [37, 47].

The statistical power is defined as the probability of rejecting the null hypothesis, i.e., the probability of finding an absence of significant effect whereas one actually exists [48]. In a reference study, Cohen [49] has reported that significant differences will have a power greater than 80 %. In our study population was small but sufficient to obtain substantial relative reliability values. An a posteriori calculation showed that the power achieved by all significant results was above 97 %. Still, significant differences might have been undetected due to the relative small population size (lack of adequate power for detecting a true difference of a meaningful magnitude). The relative reliability was assessed over 14 lumbar locations and estimated for 3 distinct low back regions ((1) the left low back, (2) the right low back and (3) the overall low back). The first important result of this study using an electronic pressure algometer was that ICC ranged from 0.90 to 0.99 and were almost perfect regardless of the conducted comparisons (trial 1 vs. trial 2, trial 1 vs. trial 3, trial 2 vs. trial 3). This finding is in accordance with the existing literature when PPTs were assessed in other anatomical locations. For instance, indeed, Nussbaum and Downes [50] assessed PPT relative reliability in the biceps brachii muscle by means of a Fischer algometer using 3 consecutive trials over 3 consecutive days. Nussbaum and Downes [50] have also reported excellent reliability regardless of the comparison considered (trial 1 vs. trial 2, trial 2 vs. trial 3 and trials 1 vs. trial 2 vs. trial 3). In 2011, Walton and colleagues [35] have tested intra-rater reliability with 3 consecutive PPT measurements on 2 anatomical locations (trapezius and tibialis anterior) with an electronic pressure algometer and reported excellent ICC values (0.96 and 0.97). However, the authors have only compared the mean between the second and the third PPT assessment. A comparison between two consecutive assessments is common in studies dealing with PPT but questions the rationale behind the fact that three PPT values are often recorded [33, 37, 51].

Farasyn and Meeusen [30] have calculated ICC of three consecutive PPT measurements on erector spinae muscles with a Fischer algometer on healthy volunteers and have also compared these PPT measurements by series of two and have reported excellent relative reliability. In contrast to our findings, the PPT of the initial trial has been reported to be significantly higher than the second and the third trial [30]. A similar finding has also been obtained by Lacourt and colleagues [52] that have suggested that the first trial measured using an electronic algometer should be considered as a practice trial and excluded from the analysis. Conversely, Chesterton and colleagues [32] and Nussbaum and Downes [50] using respectively an electronic and a Fisher algometer have reported that the highest ICC values are obtained when the mean score of three trials is used. Our results are different from the above mentioned works in two aspects: (1) they showed that the mean of the three trials generated ICC and SEM values identical to those generated by 2 consecutive measurements (Trials 1–2 or Trials 1–3 or Trials 2–3); and (2) they showed that the first measurement did not generate higher PPT values than the second or third assessment. In other words, the first measurement does not necessarily need to be considered as a practice one and can thus be taken into account for analysis when assessing PPT values over the low-back region of vine-workers. However, this result is in accordance with a recent study assessing PPT over the low back of healthy individuals suggesting not to discard the first trial to report higher PPT reliability [31]. Furthermore, this finding highlights the importance of the experimental procedure, namely, the information given to the workers and the relevance of the familiarization PPT assessments on e.g. a remote body part like the tibialis anterior muscle in the present study.

Although numerous studies have assessed the relative reliability of PPT, only few studies have investigated actually reported the absolute reliability making difficult comparison among the existing literature. The reported SEMs were similar to what has been recently reported by Madeleine and colleagues [33] after a test-retest on the erector spinae muscle of young football players using an electronic algometer (i.e. 60.4 kPa). In other anatomical locations like the upper trapezius and tibialis anterior, Walton and colleagues [35] have obtained SEM ranged from 18.2 to 73.8 kPa while Chesterton and colleagues [32] have found SEM of approx. 60 kPa for the first dorsal interosseous muscle (both using an electronic algometer). We found no statistical differences between SEM regardless of the conducted comparisons between trials except for the comparison Trials 2–3 vs. Trials 1–3 which suggests that the first assessment did not generate an error superior to other two.

With respect to MDC, we notice that the reported values were regularly above 150 kPa which is still in accordance with existing literature. Fischer [53], Chesterton and colleagues [32] and Madeleine and colleagues [33] have also reported MDC values above 100 kPa. This implies a small sensitivity to change and that a change in PPT measurement can be masked by the measurement error regardless of the absolute changes in PPT due to an ergonomics intervention [33].

### Limits and perspectives

This study presents several limitations. Although many studies have demonstrated that inter-rater [33, 49] and test-retest reliability are excellent on pain free subjects, our knowledge on population of vine-workers suffering from musculoskeletal pain is still limited. Individual factors like gender, age, BMI and the intensity of pain affect PPT [36, 54–56]. The studied population was composed of men and women, different age, BMI and pain intensity. Consequently, we conducted new analyses using a median split [54]. In line with literature, we found lower PPT values for women and workers reporting pain above 2.5 on a VAS [36, 56]. However, the computed ICC, SEM and MDC remained similar to values found for the entire population. Future studies assessing PPTs values between pain-free workers and workers suffering from WMSDs adjusted for individual factors are thus warranted. Although no link between PPTs and risk of future low back pain and no PPT differences among workers with or without recurrent low back pain have been reported [47, 57], a larger sample size could help to establish a set of normative PPTs’ values for vine-workers [20]. Finally, since PPT is increasingly used to assess the effect of physical training on pain sensitivity [58] or to monitor the effectiveness of an intervention [33, 59, 60], PPT could be used as a pain biomarker among vine-workers to assess over time the effects of ergonomic interventions or physical training programs specifically designed to prevent WMSDs.

## Conclusions

The present study showed PPTs assessed over the low back region of vine-workers have excellent relative and absolute reliability. Reliable PPT assessments can be equally achieved when using the mean of three PPT measurement or with the first measurement. The relative and absolute reliability remained similar to the ones obtained for the entire population when taking gender, age, BMI and pain intensity in consideration but PPT were lower for women and in presence of pain. The findings suggest that the assessment of PPT over the low back region of vine-workers can be used to measure the effects of interventions.

## Abbreviations

ANOVA: Analysis of variance
BMI: Body mass index
CI: Confidence interval
ICC: Intraclass correlation coefficient
LOA: Limit of agreement
MDC: Minimum detectable change
NRS: Numeric rating scale
PPT: Pressure pain threshold
RM: Repeated measure
SD: Standard deviation
SEM: Standard error of the mean
VAS: Visual analog scale
WMSD: Work related musculo-skeletal disorders
